# Structure–Activity Relationship Study Reveals the Molecular Basis for Specific Sensing of Hydrophobic Amino Acids by the *Campylobacter jejuni* Chemoreceptor Tlp3

**DOI:** 10.3390/biom10050744

**Published:** 2020-05-11

**Authors:** Mohammad F. Khan, Mayra A. Machuca, Mohammad M. Rahman, Cengiz Koç, Raymond S. Norton, Brian J. Smith, Anna Roujeinikova

**Affiliations:** 1 Infection and Immunity Program, Monash Biomedicine Discovery Institute, Clayton, Victoria 3800, Australia; mohammad.khan@monash.edu (M.F.K.); maymaper@uis.edu.co (M.A.M.); mohammad.mizanur.rahman@monash.edu (M.M.R.); cengiz.koc.khan@gmail.com (C.K.); 2 Department of Microbiology, Monash University, Clayton, Victoria 3800, Australia; 3 Medicinal Chemistry, Monash Institute of Pharmaceutical Sciences, Monash University, Parkville, Victoria 3052, Australia; ray.norton@monash.edu; 4 ARC Centre for Fragment-Based Design, Monash University, Parkville, Victoria 3052, Australia; 5 La Trobe Institute for Molecular Science, La Trobe University, Melbourne, Victoria 3086, Australia; brian.smith@latrobe.edu.au; 6 Department of Biochemistry and Molecular Biology, Monash University, Clayton, Victoria 3800, Australia

**Keywords:** *Campylobacter jejuni*, chemoreceptor, methyl-accepting chemotaxis protein, dCache, high-throughput screening

## Abstract

Chemotaxis is an important virulence factor of the foodborne pathogen *Campylobacter jejuni*. Inactivation of chemoreceptor Tlp3 reduces the ability of *C. jejuni* to invade human and chicken cells and to colonise the jejunal mucosa of mice. Knowledge of the structure of the ligand-binding domain (LBD) of Tlp3 in complex with its ligands is essential for a full understanding of the molecular recognition underpinning chemotaxis. To date, the only structure in complex with a signal molecule is Tlp3 LBD bound to isoleucine. Here, we used in vitro and in silico screening to identify eight additional small molecules that signal through Tlp3 as attractants by directly binding to its LBD, and determined the crystal structures of their complexes. All new ligands (leucine, valine, α-amino-N-valeric acid, 4-methylisoleucine, β-methylnorleucine, 3-methylisoleucine, alanine, and phenylalanine) are nonpolar amino acids chemically and structurally similar to isoleucine. X-ray crystallographic analysis revealed the hydrophobic side-chain binding pocket and conserved protein residues that interact with the ammonium and carboxylate groups of the ligands determine the specificity of this chemoreceptor. The uptake of hydrophobic amino acids plays an important role in intestinal colonisation by *C. jejuni*, and our study suggests that *C. jejuni* seeks out hydrophobic amino acids using chemotaxis.

## 1. Introduction

*Campylobacter jejuni* is a Gram-negative, microaerophilic, flagellated bacterium that colonises the intestines of many wild and domestic animals, resulting in a commensal relationship between the bacterium and the host [1,2,3,4]. Direct contact with infected animals or consumption of contaminated water or food can result in human infection leading bacterial gastroenteritis [5,6,7,8,9], which manifests as fever, severe abdominal pain and diarrhoea [10]. Important post-infectious complications in humans include reactive arthritis, neuromuscular paralysis, myositis, and idiopathic peripheral neuropathy [11,12].

The molecular mechanisms implicated in *C. jejuni* pathogenesis are not well understood. However, chemotaxis is known to play an important role in intestinal colonisation in avian and mammalian hosts [13,14]. Non-chemotactic *C. jejuni* mutants show a reduced ability to colonise the gastrointestinal tract of mice [15,16] and reduced infectivity compared to wild-type bacteria [17]. Chemotaxis enables bacteria to find optimal niches for their proliferation by moving towards nutrients, or away from harmful compounds. Small molecules in the environment can be sensed as attractants or repellents by a repertoire of chemoreceptors termed methyl-accepting chemotaxis proteins (MCPs) or transducer-like proteins (Tlps) [18,19,20]. Most of the characterised MCPs are membrane-embedded proteins comprised of an extracytoplasmic ligand-binding domain (LBD), two transmembrane helices, a HAMP (histidine kinases, adenylyl cyclases, methyl-accepting protein, and phosphatases) domain and a cytoplasmic methyl-accepting domain.

*C. jejuni* possesses at least 13 different Tlps [18,21,22,23,24] and exhibits chemotactic responses to amino acids, carbohydrates, organic acids, and constituents of bile and mucus [25,26,27]. Only some of the chemical signals were matched to a specific Tlp. Tlp1 has been shown to sense aspartate [28], whereas Tlp3 appears to sense multiple ligands (directly or indirectly), which gave it its other name, *Campylobacter* chemoreceptor for multiple ligands (CcmL) [29,30]. Tlp4 was identified as a sodium deoxycholate receptor [30], Tlp7 as a formic acid receptor [17], and Tlp11 as a galactose receptor [26,31].

Analysis of the crystal structure of the LBDs of *C. jejuni* Tlp1 and Tlp3 revealed that they have a double Cache (dCache; calcium channels and chemotaxis receptors) fold [32,33]. dCache domains consist of two tandem subdomains (termed membrane-distal and membrane-proximal) that likely emerged through tandem segmental DNA duplication. The dCache LBD is one of the most abundant sensing modules in prokaryotes [34]. Chemoreceptors with a dCache LBD are diverse in their ligand preferences; characterised examples include chemoreceptors that sense amino acids [28,32,33,35,36,37,38,39,40,41,42,43], organic acids related to the tricarboxylic acid (TCA) cycle [44], γ-aminobutyric acid [39,42], polyamines [45], galactose [26], and histamine [46].

dCache sensing domains recognise their cognate ligands directly or indirectly. Small molecules that are recognised directly typically bind to the membrane-distal subdomain [32,41,43,45], although direct sensing via the membrane-proximal subdomain has also been observed (*Helicobacter pylori* TlpC/lactate) [44]. It is, therefore, believed that dCache modules can employ either the membrane-distal or the membrane-proximal subdomain, or both, for ligand recognition. Indirect sensing is thought to be mediated by substrate-binding proteins; it has been suggested, for example, that *Bacillus subtilis* McpC [36] and *C. jejuni* Tlp1 [28] sense amino acids indirectly, as the amino acid ligands did not bind to their isolated LBDs.

Previous chemotactic assays with the isogenic ∆*tlp3 C. jejuni* mutant suggested that a variety of small molecules signal through Tlp3: isoleucine, purine, aspartic acid, malic acid, fumaric acid, and sodium deoxycholate signal as attractants, while lysine, glucosamine, succinic acid, arginine, and thiamine signal as repellents [29,30]. Prior to this study, the only reported crystal structure of Tlp3 LBD in complex with a signal molecule was that of isoleucine-bound Tlp3 LBD [32]. That study demonstrated that the Tlp3 side chains that form interactions with the ammonium and carboxylate groups of isoleucine (Lys149, Trp151, Tyr167, Asp169, and Asp196) are essential for isoleucine binding [32]. These five residues are structurally conserved among dCache-type chemoreceptors that are specific to amino acids [32,42,43], suggesting that the dCache-type receptors share a common mechanism of recognition of the invariant moiety of an amino acid. Furthermore, the crystal structures of dCache LBDs from various species in complex with different amino acids revealed that ligands bind to their membrane-distal subdomains in a mode that is very similar to that observed for isoleucine bound to Tlp3 LBD [42,43]. Cumulatively, these findings corroborated the notion that *C. jejuni* Tlp3 evolved to directly recognise amino acids. An important question that remains to be answered is how others reported, chemically diverse molecules, including purine, sodium deoxycholate, and glucosamine, signal through Tlp3. A previous study suggested there may be direct binding to Tlp3, but did not support this hypothesis by site-directed mutagenesis data and conceded that there was an inconsistency between the results of different binding assays [29].

To determine the ligand-binding preference of Tlp3, and to understand the structural basis behind its specificity, we performed an in vitro and in silico high-throughput screening for small molecules that bind to Tlp3 LBD. We demonstrated that all the ligands identified elicit an attractant response in *C. jejuni*, and that they signal through Tlp3. We then determined the crystal structures of their complexes and performed a structure–activity relationship (SAR) study that identified molecular features recognised by this chemoreceptor.

## 2. Methods

### 2.1. In Silico Screening

#### 2.1.1. Preparation of Ligand Library

The ligand data for all commercially available compounds with molecular weight (MW) < 300 and partition coefficient (logP < 3) were downloaded from the ZINC database [47,48,49] (1,487,000 ligands, access date 5 May 2017). A virtual library comprising 2595 L-isoleucine analogues for in silico screening was compiled following a substructure search against these data using OpenBabel [50], with isoleucine as a query substructure. Charge assignment and geometry optimisation using the Universal Force Field (UFF) [51] were carried out in OpenBabel within PyRx 0.8 [52].

#### 2.1.2. Virtual Screening for New Tlp3 LBD Ligands 

The virtual library was screened by docking into the known binding site in the membrane-distal subdomain, using the protein coordinates of Tlp3 LBD in crystal complex with L-isoleucine (PDB code: 4XMR) [32]. The docking was performed in AutoDock Vina using a flexible ligand/rigid receptor protocol [53]. The protocol was first validated and optimised by re-docking isoleucine and assessing the quality of fit between the predicted and the experimentally observed positions. Using a 25 × 25 × 25 Å box centred at the binding site resulted in the optimal docking performance (the root mean square error (RMSE) of 0.9 Å for all non-hydrogen atoms). The docking results for the 2595 isoleucine analogues were sorted according to the predicted free energy of binding, and the solubility in water of the top hits was estimated using MarvinSketch [54]. The top three water-soluble compounds (4-methyl-L-isoleucine, β-methyl-L-norleucine and 3-methyl-L-isoleucine) were selected for biophysical and structural studies (Table 1).

### 2.2. Thermal Shift Assays

Tlp3 LBD from *C. jejuni* strain NCTC 11168 was expressed in *E. coli,* purified according to the previously published procedure [55] and used for high-throughput screening against the compounds from the Biolog Phenotype Microarray (PM) libraries PM1, PM3B, and PM5 using thermal shift assays [41]. The libraries comprise potential metabolic substrates, including carbon and nitrogen sources and nutrient supplements [56]; they proved useful for identifying ligands of chemoreceptors in previous studies [55]. Each plate contains 95 compounds and a negative control (water). Thermal-shift-based screening is based on the phenomenon that ligand-binding alters thermal stability of the protein, resulting in an increase or decrease in the protein’s melting temperature (T_m_) [57,58]. Screening was performed by following the protocol previously described by McKellar et al. [41], with minor modifications. The compounds in the Biolog PM plates were dissolved in 50 μL of water, yielding a final concentration of 10–20 mM (as indicated by the manufacturer). Each assay was performed in a 25 μL volume and contained 20 μM protein, 0.8–1.6 mM Biolog compound, 10 mM Tris-HCl pH 8.0, 150 mM NaCl, and 10× SYPRO orange reagent (5000× stock, catalogue number S5692; Sigma-Aldrich). The samples were heated from 35 °C to 80 °C at a ramp rate of 0.5 °C min^−1^ using a Rotor-Gene Q real-time PCR cycler (Qiagen, Venlo, Netherlands). Protein denaturation was monitored by following changes in the SYPRO Orange fluorescence emission (λ_ex_ 530 nm/λ_em_ 555 nm) that result from the dye binding to the exposed hydrophobic protein regions. All experiments were performed in triplicate. The midpoint of denaturation (the melting temperature T_m_) was calculated by fitting the data to the Boltzmann sigmoidal equation for the two-state unfolding model using GraphPad Prism (version 7.02) (GraphPad Software, La Jolla, CA, USA). The thermal shift assay with the three potential ligands identified using in silico screening was performed at a ligand concentration of 5 mM, with other conditions the same.

### 2.3. Isothermal Titration Calorimetry (ITC)

Tlp3 LBD was dialysed against a buffer containing 100 mM Tris-HCl pH 8.0 and 150 mM NaCl. The candidate ligands were dissolved in the dialysis buffer, at a concentration of 3 mM for L-leucine, α-amino-N-valeric acid, 4-methyl-L-isoleucine, β-methyl-L-norleucine and 3-methyl-L-isoleucine, 5 mM for L-phenylalanine, 10 mM for L-valine, 2’-deoxycytidine, Ala-Thr, methyl pyruvate, L-lysine, L-arginine, L-aspartate and purine, and 50 mM for L-alanine. Calorimetry measurements were conducted on a VP-ITC MicroCal calorimeter (Malvern Instruments, UK) at 25 °C. The Tlp3 LBD sample (10 µM) in a 1.45 mL reaction cell was titrated with 25 successive 10 µL injections of ligand solution at a spacing of 200 s. All measurements were made in triplicate and corrected for heat changes measured when the ligand was titrated into the buffer. The data were integrated and normalised for protein concentration to generate binding isotherms. The isotherms were fitted to a single-site binding model (N = 1) in Origin 7 (OriginLab, Northampton, MA, USA) using nonlinear least-squares regression, to calculate the binding enthalpy ∆H, dissociation constant *K*_d_, and binding entropy ∆*S*.

### 2.4. Construction of Isogenic Δtlp3 Mutant in C. Jejuni NCTC 11168

A Δ*tlp3*::*kan* allele was generated by replacing the open reading frame (ORF) of wild-type (WT) *tlp3* of *C. jejuni* strain NCTC 11168 with the *C. coli* kanamycin resistance gene *aphA3* (795 bp, Genbank ID HG515011.2) [59]. A DNA fragment containing the *aphA3* ORF, flanked by *C. jejuni* chromosomal DNA sequences corresponding to the 721 bp upstream and 701 bp downstream of the *tlp3* ORF (locus tag Cj1564, Genbank ID AL111168.1), was synthesised and ligated into the pUC18 vector by GenScript (USA, Figure S1A). The resulting plasmid was used to transform *C. jejuni* NCTC 11168 by electroporation using a protocol adapted from [60]. Cells were streaked onto brain–heart infusion (BHI) agar supplemented with 5% (*v*/*v*) defibrinated horse blood and 8 µg mL^−1^ trimethoprim and grown overnight at 37 °C under microaerophilic conditions generated using the CampyGen (Oxoid) system, after which they were re-streaked onto fresh BHI-blood agar and grown for a further 18 h. Cells were then harvested in BHI broth, washed three times with an ice-cold buffer containing 272 mM sucrose and 15% (*v*/*v*) glycerol, and resuspended in the wash buffer to an optical density of 0.5 at 600 nm. Briefly, 15 µg of plasmid DNA in sterile water was added to 100 µL of resuspended cells, and the sample was electroporated at 1.8 kV, 250 Ω, 25 µF in a BioRAD Gene Pulser cuvette (0.2 cm gap) using a BTX ECM630 exponential decay wave electroporation system. The cells were recovered by flushing the cuvette with 200 µL Hanahan’s broth [61] (20 g L^−1^ tryptone, 5 g L^−1^ yeast extract, 2.4 g L^−1^ MgSO_4_, 0.5 g L^−1^ NaCl, 0.186 g L^−1^ KCl), and the suspension was pipetted onto a BHI-blood agar plate supplemented with trimethoprim. After 5 h incubation to allow for expression of *aphA3*, cells from the recovery plate were harvested and streaked on a fresh BHI-blood agar plate, supplemented with 8 µg mL^−1^ trimethoprim and 50 µg mL^−1^ kanamycin. Single colony screening for a double-crossover homologous recombination event was performed by PCR (Figure S1B), and the *C. jejuni* Δ*tlp3*::*aphA3* mutant (hereafter referred to as Δ*tlp3* mutant) was further confirmed by Sanger sequencing.

### 2.5. Chemotaxis Assay

*C. jejuni* WT strain was grown under microaerophilic conditions at 37 °C for 48 h on Columbia blood agar (CBA, Oxoid) supplemented with 5% (*v*/*v*) defibrinated horse blood plus an antibiotic cocktail comprising 2.5 U mL^−1^ polymyxin B, 2.5 µg mL^−1^ trimethoprim and 10 µg mL^−1^ vancomycin (PTV, all antibiotics from Sigma-Aldrich). The mutant strain (Δ*tlp3*) was grown on plates supplemented with 10 µg mL^−1^ kanamycin (Thermo Fisher Scientific, PTV-K). Several colonies were inoculated into Brucella broth (Becton Dickinson) using a sterile cotton swab and grown overnight with agitation on an orbital shaker set at 120 rpm min^−1^. The cells were harvested by centrifugation and resuspended in phosphate-buffered saline (PBS) to an OD_600_ of 1.2.

The nutrient-depleted chemotaxis assay was performed using the hard agar plug (HAP) method [28]. HAPs were prepared according to the method described in [62] with minor modifications. Each tested compound was dissolved in 7.5 mL of PBS at concentrations between 50 and 200 mM, depending on its solubility, and mixed with the equivalent volume of molten 4% (*w*/*v*) bacteriological agar (Oxoid) prepared in PBS. The mixture was immediately poured into a 90 mm × 14 mm Petri dish, allowed to solidify, and then HAPs (4 mm in diameter) were cut using a sterile disposable Pasteur pipette. HAPs containing the known chemoattractant serine (100 mM) or PBS were used as a positive and background controls, respectively.

To prepare plates for chemotaxis assays, HAPs containing the tested compounds were placed into Petri dishes (90 mm × 14 mm) in a circular arrangement, approximately 1 cm from the edge of the plate. Then, 7.5 mL of the bacterial cell suspension in PBS was mixed with 7.5 mL of 0.6% (*w*/*v*) bacteriological agar in PBS tempered at 50 °C, and the mixture was poured into the Petri dish with HAPs. Plates were dried at room temperature for 15 min and incubated at 37 °C for 24 h under microaerophilic conditions to allow bacteria to migrate along the chemical gradients. To enumerate bacteria in the regions around the HAPs, round agar pieces (8 mm in diameter) were cut out together with the HAPs at their centre, vortexed in 1 mL of Brucella broth, and incubated with shaking for 1 h at 37 °C under microaerophilic conditions. The sample was then diluted serially and plated onto CBA-blood plates supplemented with PTV (for the WT strain) or PTV-K (for the mutant strain) to perform a viable cell count. Chemotaxis assays were performed in triplicate.

The chemotactic index (CI) was calculated as the ratio of the number of cells recovered from the area around the HAP containing the tested compound to the number of cells recovered from the area around the chemotactically neutral control (PBS). CI > 1 is indicative of chemoattractant response, whereas a value of <1 represents chemorepellent response.

### 2.6. Protein Crystallisation, Data Collection and Structure Determination

Prior to crystallisation, Tlp3 LBD was concentrated to 15 mg mL^−1^ in a buffer containing 10 mM Tris-HCl pH 8.0 and 150 mM NaCl, and incubated with 8 mM ligand (L-leucine, L-valine, α-amino-N-valeric acid, L-lysine, L-aspartate, L-arginine, malic acid, 4-methyl-L-isoleucine, β-methyl-L-norleucine, 3-methyl-L-isoleucine, L-alanine or L-phenylalanine) for 30 min at room temperature. The solution was then clarified by centrifugation at 13,000 *g*, 4 °C for 20 min and protein crystallisation was carried out by the hanging drop vapour diffusion method using crystallisation conditions similar to those described previously [63]. Briefly, crystals were grown in 2 µL hanging drops suspended over 500 µL reservoir solution containing 22% (*w*/*v*) polyethylene glycol 3350, 100 mM sodium citrate pH 5.0 and 200 mM ammonium sulphate. The hanging drop was prepared by adding 1 µL of the protein/ligand mix to 1 µL of the reservoir solution. Crystals of all Tlp3 LBD/ligand complexes had similar unit-cell parameters and belonged to space group *P*2_1_ (Table 2), with two molecules in the asymmetric unit.

To perform data collection at cryogenic temperatures, the crystals were briefly soaked in a cryoprotectant solution consisting of 26% (*w*/*v*) polyethylene glycol 3350, 100 mM sodium citrate pH 5.0, 200 mM ammonium sulphate, 10% (*v*/*v*) glycerol, and 10 mM respective ligand, and then flash-cooled by plunging them into liquid nitrogen. X-ray diffraction data were collected at 100 K on the MX1 and MX2 beamlines of the Australian Synchrotron [64]. The data were processed using iMOSFLM [65] and scaled with AIMLESS [66] from the CCP4 software suite [67]. A summary of the data processing statistics is presented in Table 2.

The crystal structures were determined using molecular replacement with PHASER [68] and the protein coordinates of the Tlp3 LBD/isoleucine complex (PDB code, 4XMR) [32] as a search model. The models were refined with PHENIX [69] and manually rebuilt where necessary using COOT [70]. Analysis of the stereochemical quality of the final models was performed using MOLPROBITY [71]. The refinement statistics are summarised in Table 3. The accessible surface area was calculated using AREAIMOL from CCP4. The structure figures were produced using PyMOL 4.1 [72].

### 2.7. RCSB PDB Accession Numbers

The coordinates and structure factors of the Tlp3 LBD/ligand complexes obtained in this study were deposited in the RCSB PDB under accession codes (PDB IDs) 6W3S (complex with L-leucine), 6W3X (L-valine), 6W3T (α-amino-N-valeric acid), 6W3O (4-methyl-L-isoleucine), 6W3P (β-methyl-L-norleucine), 6W3R (3-methyl-L-isoleucine, 6W3Y (L-alanine), and 6W3V (L-phenylalanine).

## 3. Results

### 3.1. Screening for Potential Tlp3 LBD Ligands by Thermal Shift Assays

Compounds that serve as carbon and nitrogen sources for bacteria are often sensed as attractants; we hypothesised that some of the ligands that signal through Tlp3 by directly binding may belong to this class. We, therefore, performed fluorescence-based thermal shift assays to screen recombinant Tlp3 LBD against 248 such compounds in the Biolog Phenotype Microarray (PM) libraries PM1, PM3B, and PM5. PM screens were originally designed to characterise the metabolic capabilities of microbial species, but as they include a very broad range of chemicals, they also proved useful as a library of potential ligands to probe the specificity of chemoreceptors [41,73,74].

**Figure 1:**
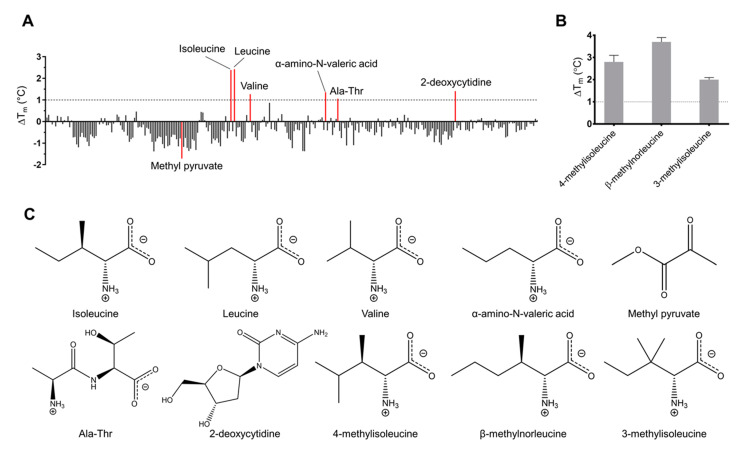
Results of thermal shift assay-based screening of recombinant Tlp3 LBD against (**A**) Biolog small molecule libraries PM1, PM3B, and PM5, and (**B**) candidate ligands identified by in silico screening. The ΔTm threshold above which the small molecule was considered to be a potential binder was set at 1.0 °C. The compound that caused the most significant destabilisation of the protein (methyl pyruvate, ΔT_m_ = −1.8 °C) was also selected for further testing as a potential binder. (**C**) Structures of all new potential Tlp3 LBD ligands identified by screening against PM libraries and by in silico screening, drawn in MarvinSketch [54].

The midpoint of unfolding (T_m_) of free Tlp3 LBD was 56.3 ± 0.1 °C. The ability of the tested compounds to alter the thermal stability of the protein was quantified by the thermal shift they induced (ΔT_m_ = T_m_ (in the presence of the compound) − T_m_ (free protein)). The results of the screening are presented in Table S1 and summarised in Table 4. The known ligand L-isoleucine induced a thermal shift of 2.1 °C; we, therefore, set the ΔT_m_ threshold, above which the small molecule was considered as a potential binder, to 1.0 °C. Apart from L-isoleucine, we identified a total of five compounds that increased the T_m_ value by at least 1.0 °C (Figure 1A, Table 4 and Table S1). L-leucine induced the highest thermal shift (ΔT_m_ = 2.4 °C), followed by 2-deoxycytidine, α-amino-N-valeric acid, L-valine, and Ala-Thr, which induced thermal shifts of 1.4 °C, 1.4 °C, 1.3 °C, and 1.1 °C, respectively. Furthermore, we observed that many of the tested molecules decreased the T_m_ (i.e., reduced the stability of the protein, Figure 1A, Table S1), with methyl pyruvate causing the most significant destabilisation of the protein (ΔT_m_ = −1.8 °C). At that stage, methyl pyruvate was also selected as a potential binder.

We note that the small molecule library we screened included seven of the 11 compounds that were previously reported to signal through Tlp3 [29] (L-isoleucine, L-aspartic acid, malic acid, L-lysine, succinic acid, L-arginine, and thiamine). With the exception of L-isoleucine, the ΔT_m_ values for these signal molecules were negligibly low (≤0.1 °C), indicating a lack of direct binding to Tlp3 LBD (Table 4).

### 3.2. Screening for New Potential Isoleucine-Like Chemoeffectors by Molecular Docking

The results of screening by thermal shift assays suggested that, in addition to L-isoleucine, amino acids that are chemically and structurally similar to L-isoleucine, namely L-valine and α-amino-N-valeric acid, bind to Tlp3 LBD. We thus hypothesised that the hydrophobic ligand-binding pocket in the membrane-distal subdomain of Tlp3 LBD can recognise a spectrum of isoleucine-like natural and non-natural amino acids. To identify candidate signal molecules of this class, we generated a virtual library comprising ~2600 L-isoleucine analogues and screened it by docking into the known L-isoleucine binding site of Tlp3 LBD. The three water-soluble compounds with the lowest predicted free energy of binding (4-methyl-L-isoleucine, β-methyl-L-norleucine, and 3-methyl-L-isoleucine) were selected for the subsequent experiments (Table 1). In the thermal shift assay, the ΔT_m_ values for all three compounds (2.8 °C, 3.7 °C, and 2.0 °C (Figure 1B and Table 4)) exceeded the 1.0 °C threshold, suggesting their direct binding to Tlp3 LBD.

### 3.3. Ligand Binding Affinity Measurements by Isothermal Titration Calorimetry (ITC)

The binding affinities of the new potential Tlp3 LBD ligands identified by screening against the Biolog PM libraries and by in silico screening (see Figure 1C for their structures) were measured by ITC. To validate the results of the thermal shift assays, three signal molecules that did not induce any significant protein stabilisation (L-lysine, L-arginine and L-aspartate, ΔT_m_ ≤ 0.1 °C) were included as a negative control. In addition, we tested purine, which was also previously reported to signal through Tlp3 [29]. Furthermore, also for validation purpose, we quantified the Tlp3 LBD interaction with L-alanine and L-phenylalanine that, like L-isoleucine, have apolar side chains, but had no comparable effect on the protein’s stability in thermal shift assays (Table S1).

**Figure 2:**
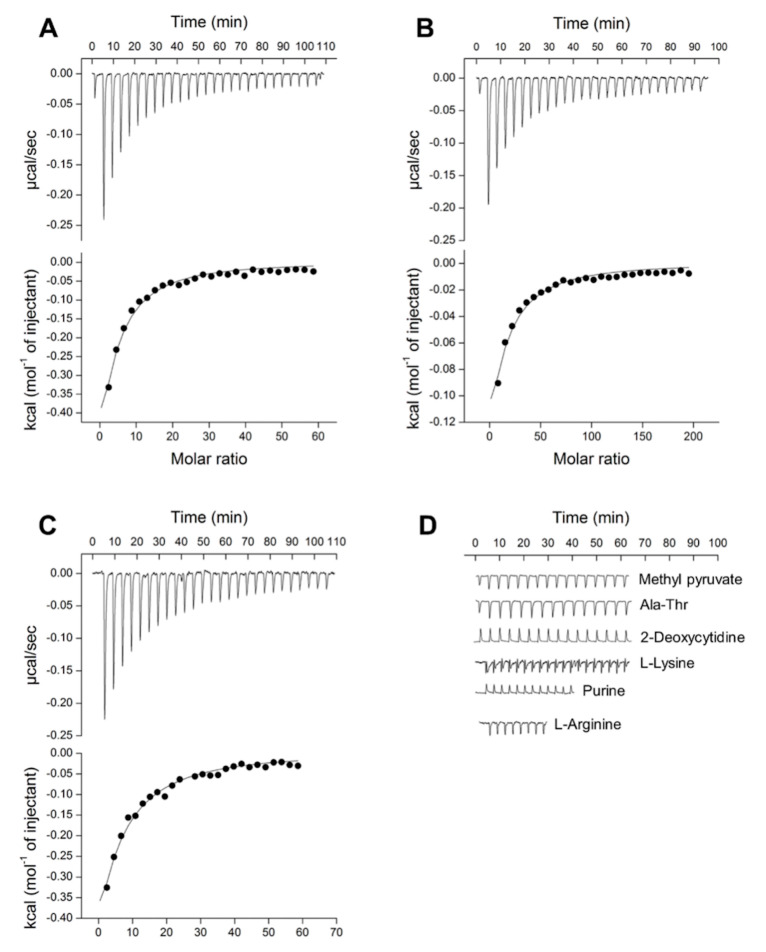
ITC titrations of Tlp3 LBD (10 μM) with (**A**) L-leucine (3 mM), (**B**) L-valine (10 mM), (**C**) α-amino-N-valeric acid (3 mM), and (**D**) methyl pyruvate, Ala-Thr, 2-deoxycytidine, L-lysine, purine, and L-arginine (each at 10 mM). The solid line represents the least-squares fit of the experimental data to a single-site binding mode.

**Figure 3:**
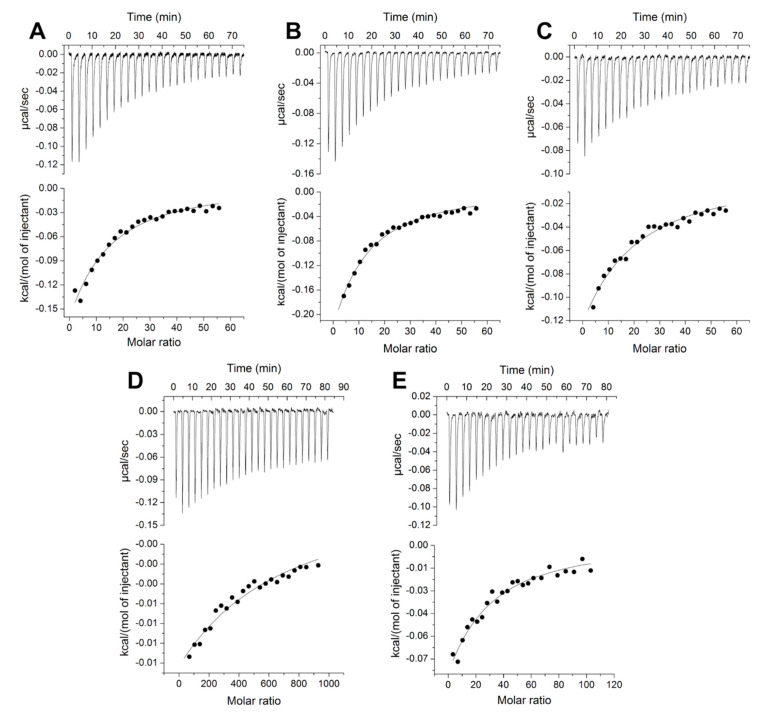
ITC titrations of Tlp3 LBD (10 μM) with (**A**) 4-methyl-L-isoleucine (3 mM), (**B**) β-methyl-L-norleucine (3 mM), (**C**) 3-methyl-L-isoleucine (3 mM), (**D**) L-alanine (50 mM), and (**E**) L-phenylalanine (5 mM). The solid line represents the least-squares fit of the experimental data to a single-site binding mode.

The results of the ITC experiments confirmed our hypothesis that, in addition to L-isoleucine, Tlp3 LBD binds natural and non-natural amino acids that are similar to L-isoleucine in structure, with apparent affinities ranging from 105 to 484 µM (Table 4, Figure 2A–D and Figure 3A–E). Among the natural amino acids, the highest affinities were measured for L-isoleucine (*K*_d_ = 86 μM [32]) and L-leucine (*K*_d_ = 105 μM), followed by L-valine (four- to five-fold weaker binding affinity compared to isoleucine and leucine). Among the non-natural amino acids, α-amino-N-valeric acid showed a slightly lower affinity for Tlp3 LBD (168 μM) than isoleucine and leucine, followed by β-methyl-L-norleucine (*K*_d_ = 294 μM), 4-methyl-L-isoleucine (*K*_d_ = 324 μM), and 3-methyl-L-isoleucine (*K*_d_ = 484 μM). The measured binding affinities of apolar amino acids that are least similar to isoleucine in structure—alanine (*K*_d_ ≈ 5 mM) and phenylalanine (*K*_d_ = 730 μM)—were markedly lower than that of isoleucine, in agreement with the results of the thermal shift assays.

Methyl pyruvate, which had a destabilising effect on Tlp3 LBD in thermal shift experiments, showed no binding to Tlp3 LBD, and neither did the dipeptide Ala-Thr or 2-deoxycytidine, the thermal shifts for which were very close to the cut-off value (Figure 1A and Figure 2D, Table 4). Furthermore, no binding between Tlp3 LBD and L-lysine, L-arginine, or L-aspartate could be detected by ITC, in line with the results of the thermal shift assays (Figure 2D, Table 4). Purine did not bind to Tlp3 LBD either (Figure 2D).

### 3.4. C. jejuni Tlp3 Mediates Positive Chemotactic Response to All Identified Ligands

**Figure 4:**
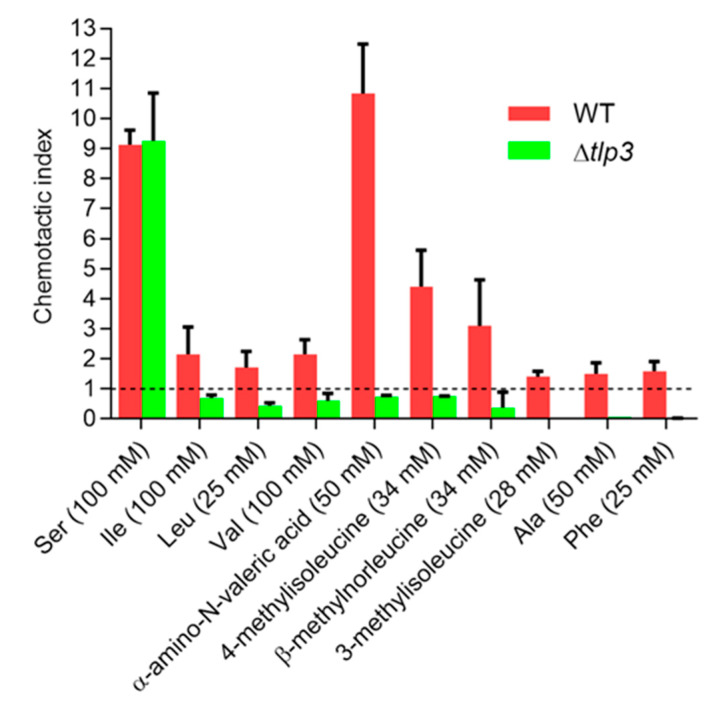
L-isoleucine analogues, L-alanine and L-phenylalanine elicit Tlp3-dependent attractant response in *C. jejuni*. Nutrient-depleted hard agar plug-based method was used. Values in the parentheses along the X-axis indicates the ligand concentrations used in the preparation of plugs; they are not identical as the standard assay concentration of 100 mM [62] could not be achieved for all ligands due to their solubility limit. L-serine and L-isoleucine were used as known Tlp3-independent and Tlp3-dependent attractant response controls, respectively. Error bars represent the standard error of the mean for three independent experiments.

To test the functional relevance of the observed specific interactions between Tlp3 and the new ligands identified in this study, we assessed the chemotactic response of *C. jejuni* to these compounds using a nutrient-depleted hard agar plug-based chemotaxis assay [28] that enumerates cells accumulated around the agar plugs containing the tested compounds. The results of this assay are presented in the form of a chemotactic index (CI) which corresponds to the number of cells accumulated around the plug containing the tested compound divided by the number of cells recovered from an equivalent size area around the plug containing a chemotactically neutral control (PBS). Chemoattractant (positive) response results in CI > 1, while chemorepellent (negative) response produces CI < 1. As illustrated in Figure 4, all ligands elicited a chemoattractant response in WT *C. jejuni*.

We next examined whether Tlp3 was required for these attractant responses. We generated an isogenic Δ*tlp3* mutant strain lacking *tlp3*; since *tlp3* is a single-gene operon, its replacement with the kanamycin resistance gene *aphA3* is not likely to have polar effects on other genes. The mutation did not affect the bacteria’s ability to respond to serine (Figure 4), a known attractant [25] that signals through an, as yet, unidentified chemoreceptor. This observation confirmed that the general signal transmission pathway of chemotaxis in the Δ*tlp3* mutant was not defective. The chemotactic responses to the new Tlp3 ligands, however, switched from attractant to repellent (Figure 4), indicating that it is Tlp3 that mediates chemoattractant response to these compounds.

### 3.5. Crystallographic Analysis of Binding Modes of New Chemoeffectors 

Tlp3 LBD has putative ligand-binding pockets in both the membrane-distal and proximal subdomains [32]. To establish whether new chemoeffectors bind to the membrane-distal subdomain, as is the case with L-isolecucine [32], or to the membrane-proximal subdomain, we determined the crystal structures of their respective complexes with Tlp3 LBD, obtained by co-crystallisation. Crystals of the complexes with L-leucine, L-valine, α-amino-N-valeric acid, 4-methyl-L-isoleucine, β-methyl-L-norleucine, 3-methyl-L-isoleucine, L-alanine, and L-phenylalanine had similar unit-cell dimensions and belonged to space group *P*2_1_ (Table 2), with a dimer in the asymmetric unit. The structures of the protein dimers from all complexes could be superposed with an average pairwise C_α_ root mean square deviation (RMSD) value of 0.23 Å, showing no significant differences. We, therefore, present the analysis of the similarities and differences in the relative positions of the respective amino acid ligands in the receptor-binding site.

**Figure 5:**
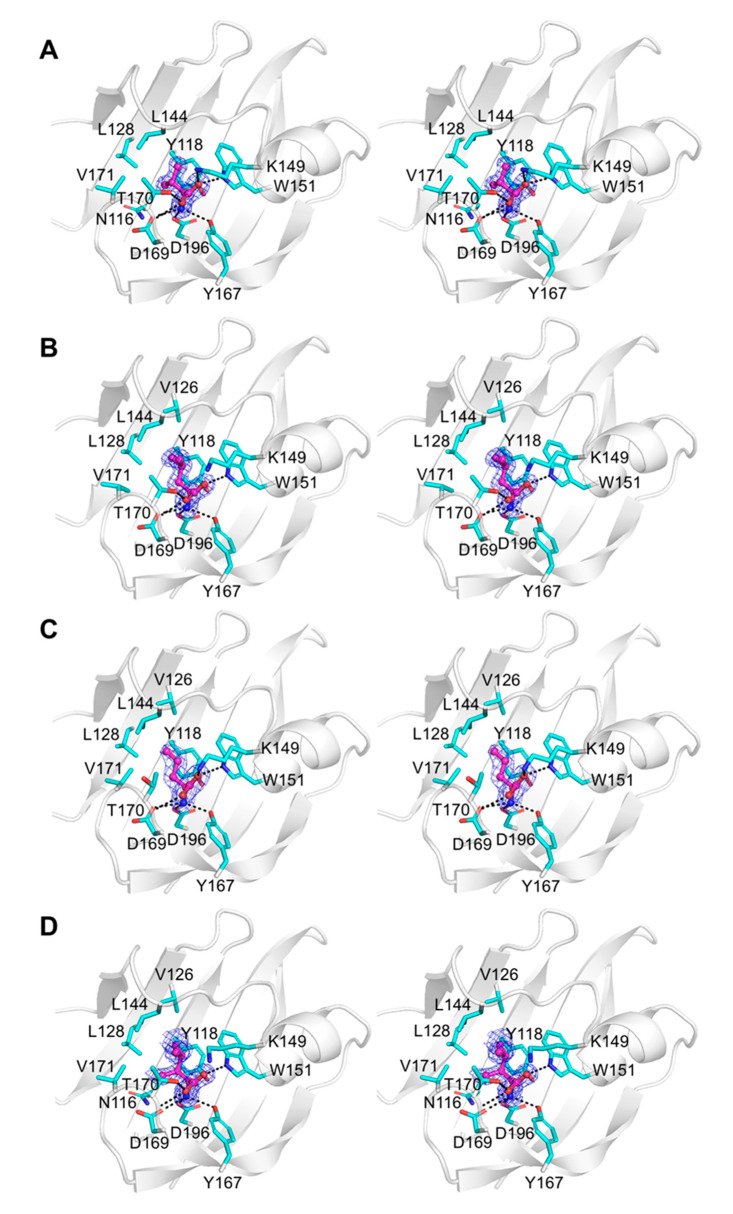
Close-up stereoview showing simulated annealing composite omit electron density for amino acids bound to membrane-distal subdomain of Tlp3 LBD in respective complexes with: (**A**) L-valine, (**B**) L-leucine, (**C**) α-amino-N-valeric acid, and (**D**) 4-methyl-L-isoleucine. The ligand molecules are shown in all-atom ball-and-stick representation with C atoms coloured purple. The protein side chains that form direct contacts with the ligands are shown in stick representation. The map was contoured at the 1.0 σ level.

**Figure 6:**
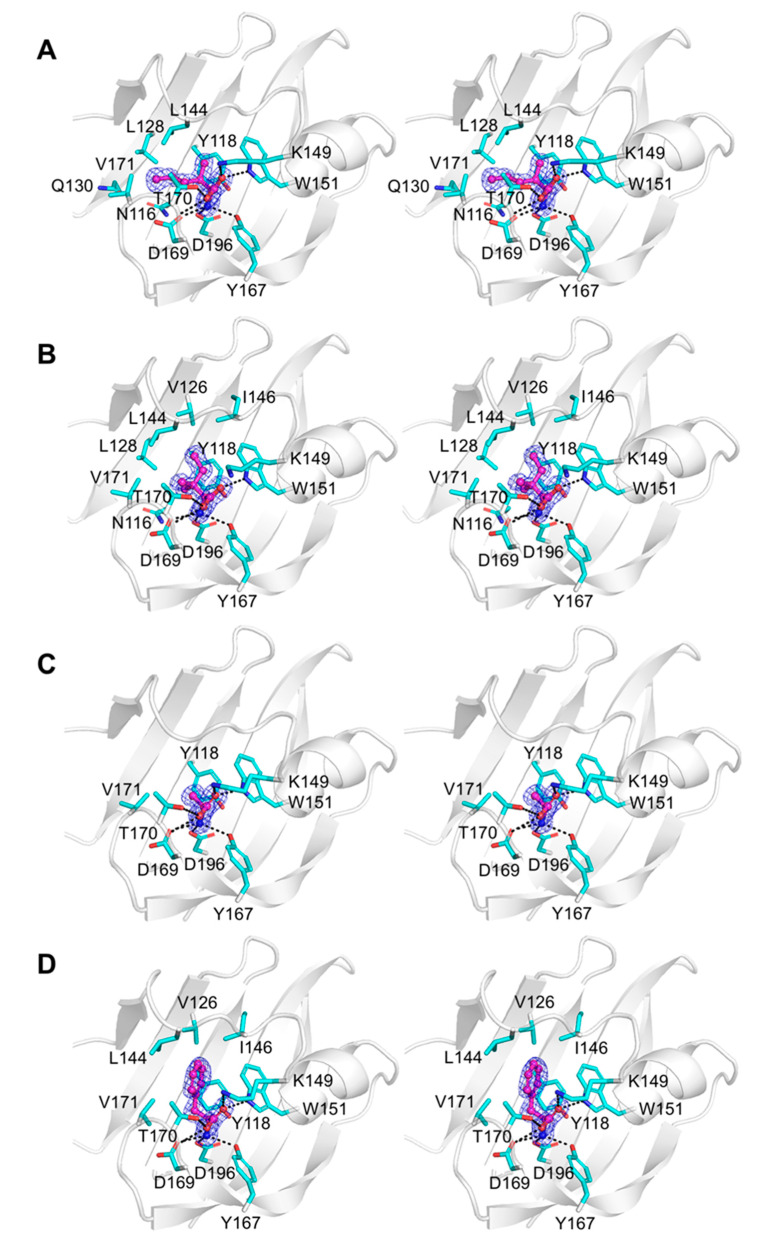
Close-up stereoview showing simulated annealing composite omit electron density for amino acids bound to membrane-distal subdomain of Tlp3 LBD in respective complexes with: (**A**) β-methyl-L-norleucine, (**B**) 3-methyl-L-isoleucine, (**C**) L-alanine, and (**D**) L-phenylalanine. The ligand molecules are shown in all-atom ball-and-stick representation with C atoms coloured purple. The protein side chains that form direct contacts with the ligands are shown in stick representation. The map was contoured at the 1.0 σ level.

**Figure 7:**
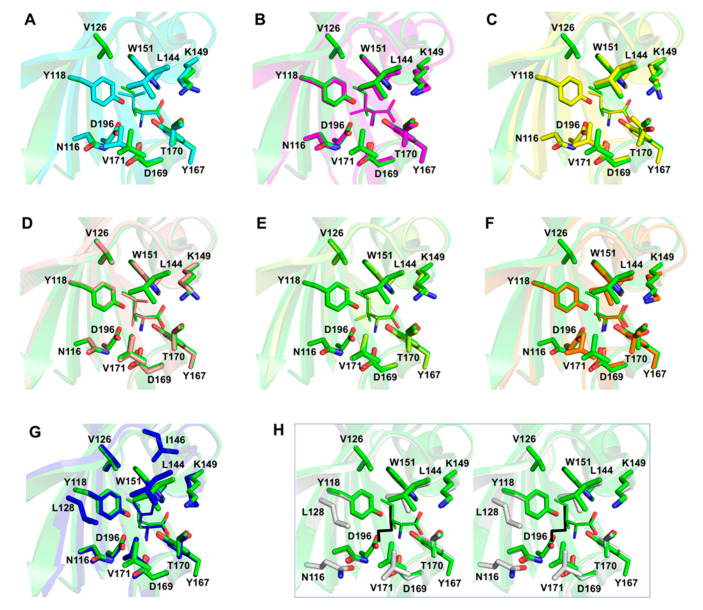
Ribbon diagram of Tlp3 LBD/isoleucine complex (green, PDB ID 4XMR) superimposed on Tlp3 LBD complexes with (**A**) leucine (cyan), (**B**) valine (magenta), (**C**) α-amino-N-valeric acid (yellow), (**D**) 4-methyl-L-isoleucine (salmon), (**E**) 3-methyl-L-isoleucine (lime), (**F**) alanine (orange), (**G**) phenylalanine (blue), and (**H**) β-methyl-L-norleucine (the latter in stereo; protein, white/black for protein/ligand). The diagram illustrates a similar mode of ligand binding. The ligands and the protein residues they contact in their respective complexes are shown in thin and thick stick representation, respectively.

The crystals of all complexes were well ordered, and we were able to calculate high-quality Fourier difference maps at 2.1 Å resolution for the complex with α-amino-N-valeric acid, and at 1.3–1.4 Å resolution for all other complexes. Analysis of these maps revealed unambiguous electron density for each ligand bound in the pocket in the membrane-distal subdomain of Tlp3 LBD (Figure 5A–D and Figure 6A–D). Comparison of the crystal structure of Tlp3 LBD/L-isoleucine complex [32] with the final refined structures of Tlp3 LBD in complex with L-leucine (Figure 7A), L-valine (Figure 7B), α-amino-N-valeric acid (Figure 7C), 4-methyl-L-isoleucine (Figure 7D), 3-methyl-L-isoleucine (Figure 7E), L-alanine (Figure 7F), L-phenylalanine (Figure 7G), and β-methyl-L-norleucine (Figure 7H) shows that the binding modes of all the new ligands are very similar to that of isoleucine. All the new ligands are completely shielded from the solvent upon binding to Tlp3 LBD, with >99% of their accessible surface area buried. The ligands ammonium and carboxylate groups are anchored into positions that are almost identical to the positions of the ammonium and carboxylate groups of isoleucine in the Tlp3 LBD/isoleucine complex [32]. These groups interact with Tlp3 LBD residues Lys149, Trp151, Tyr167, Thr170, Asp169, and Asp196 (Figure 5, Figure 6 and Figure 7), which are strongly conserved in dCache-type chemoreceptors that recognise amino acids.

The Tlp3 LBD/valine complex is stabilised by seven intermolecular hydrogen bonds (Figure 5A), most of which are present in all other ligand-bound Tlp3 LBD structures determined in this study, and the previously characterised isoleucine complex [32]. The L-valine ammonium group forms hydrogen bonds with Tyr167 O^η^, Asp169 O^δ1^, and Asp196 O^δ2^, while its carboxylate oxygen atoms form hydrogen bonds with Lys149 N^ζ^, Trp151 N^ε1^, Thr170 O^γ1^ and the main-chain amide of the same residue. The aliphatic side chain of L-valine is stabilised by van der Waals contacts with the side chains of Asn116, Tyr118, Leu144, Trp151, and Val171, and approaches within 4.2 Å of the side chain of Leu128. Similar interactions with the side chains of Tyr118, Leu128, Leu144, Trp151, and Val171 are present in the complexes with L-leucine (Figure 5B) and α-amino-N-valeric acid (Figure 5C); in comparison to L-valine, binding of these two amino acids is further favoured by the establishment of additional van der Waals contacts with the side chain of Val126. The aliphatic side chain of 4-methyl-L-isoleucine forms van der Waals contacts with the side chains of Tyr118, Trp151, and Val171 and is positioned within 4.5 Å of the side chains of Asn116, Val126, Leu128, and Leu144 (Figure 5D). The side chain of β-methyl-L-norleucine makes van der Waals contacts with the side chains of Tyr118, Leu128, Leu144, Trp151, and Val171 and approaches within 4.0 Å of the side chains of Gln130 and Asn116 (Figure 6A). In the Tlp3 LBD/3-methyl-L-isoleucine complex, the apolar side chain of the ligand makes van der Waals contacts with the side chains of Tyr118, Val126, Trp151, and Val171 and is positioned within 4.0 Å of the side chains of Asn116 and Leu128, and within 4.5 Å of the side chains of Leu144 and Ile146 (Figure 6B). The side chain of L-alanine makes van der Waals contacts with the side chains of Tyr118 and Val171 (Figure 6C). These interactions are also present in the complex with L-phenylalanine, in which the ligand’s side chain forms additional van der Waals contacts with the side chains of Val126, Leu144, Ile146, and Val171 (Figure 6D).

**Figure 8:**
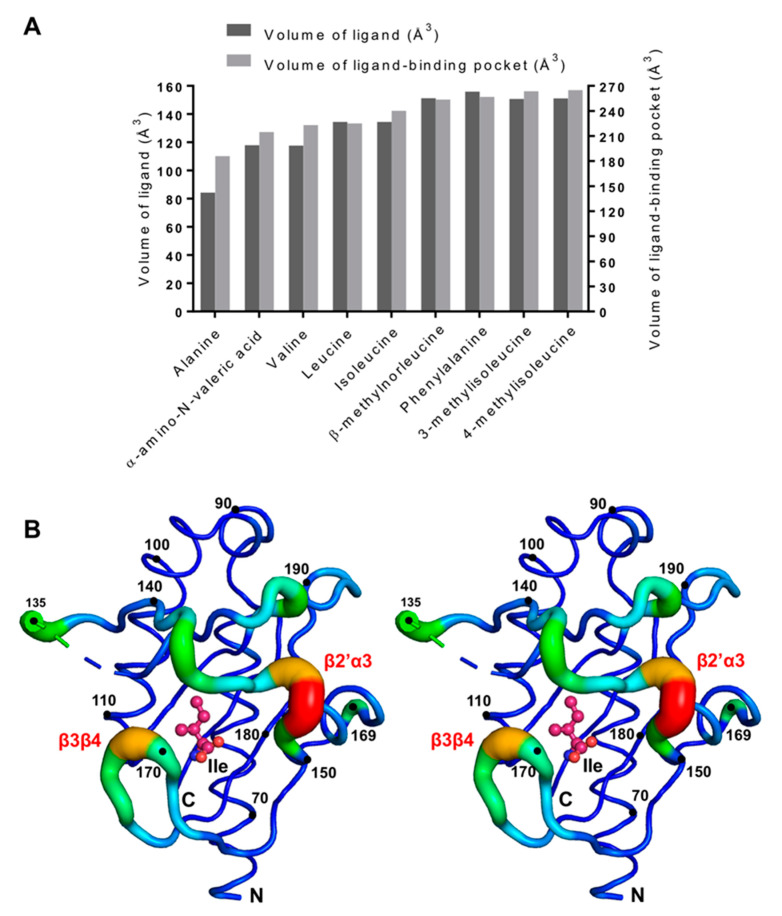
Analysis of plasticity of ligand-binding pocket in Tlp3 LBD. (**A**) Comparison of the volumes of the ligand-binding pocket of Tlp3 LBD in complexes with different ligands. It is apparent that the pocket expands according to the size of the ligand. Ligand volumes were calculated using Molinspiration (http://www.molinspiration.com/cgi-bin/properties). (**B**) Stereo diagram showing residues 63–197 of the Tlp3 LBD/isoleucine complex, drawn with the backbone radius proportional to the residue-by-residue C_α_ RMSD to the mean structure for the superimposition of membrane-distal subdomains of subunit B from all nine available Tlp3 LBD complexes. The colour gradient runs from blue (the smallest RMSD) to red (the largest RMSD). The largest displacements of Cα atoms were observed for residues Asp143 (0.65 Å), Leu144 (0.56 Å), Lys147 (0.58 Å), Thr148 (0.74 Å), and Lys149 (0.37 Å) from loop β2’α3, and Val171 (0.63 Å), Leu172 (0.40 Å), and Lys173 (0.39 Å) from β-tongue β3β4. The RMSD values were calculated using Superpose from the CCP4 suite [67].

To gain further insight into the capacity of Tlp3 to accommodate ligands of different size, we analysed the molecular surface of Tlp3 LBD in complexes with isoleucine and new apolar amino acid ligands discovered in this study, using CASTp [75] with a probe radius of 1.4 Å. This analysis revealed that the solvent-accessible volume of the ligand-binding pocket increases from a value of 186 Å^3^ for the Tlp3 LBD complex with alanine to 215 Å^3^, 223 Å^3^, 225 Å^3^, 240 Å^3^, 254 Å^3^, 257 Å^3^, 264 Å^3^, and 265 Å^3^ for the respective complexes with α-amino-N-valeric acid, valine, leucine, isoleucine, β-methylnorleucine, phenylalanine, 3-methylisoleucine, and 4-methylisoleucine (Figure 8A), providing evidence of structural plasticity of the ligand-binding site. As illustrated in Figure 8A, the pocket volume increases approximately in proportion to the size of the ligand. Comparison of the structures of all Tlp3 LBD/ligand complexes revealed that the observed variation of the volume of the ligand-binding pocket in Tlp3 LBD is achieved without significant changes in the protein structure. Calculation of the residue-by-residue C_α_ atom RMSD to the mean structure of the membrane-distal subdomain (residues 63–197) in all nine available Tlp3/LBD complexes showed that expansion/contraction of the binding pocket is largely mediated by minor changes in the backbone conformations of the two protein loops that flank it—loop β2’α3 (residues 142–148) and β-tongue β3β4 (residues 170–173, Figure 8B).

To confirm the lack of direct binding between Tlp3 and L-lysine, L-arginine, L-aspartate, or malic acid, observed in our thermal shift and isotitration calorimetry experiments, the crystal structures of Tlp3 LBD crystallised in the presence of each small molecule were also determined at pseudo-atomic resolution of 1.4, 1.45, 1.5, and 1.45 Å, respectively. The resultant electron density maps were of high quality, but revealed no electron density that could be interpreted as any of these molecules bound to Tlp3 LBD (data not shown), which was in agreement with the negative outcome of our quantitative biophysical binding studies with these candidates.

### 3.6. Analysis of Structure–Activity Relationship of Hydrophobic Amino Acids as Chemoreceptor Tlp3 Ligands

**Figure 9:**
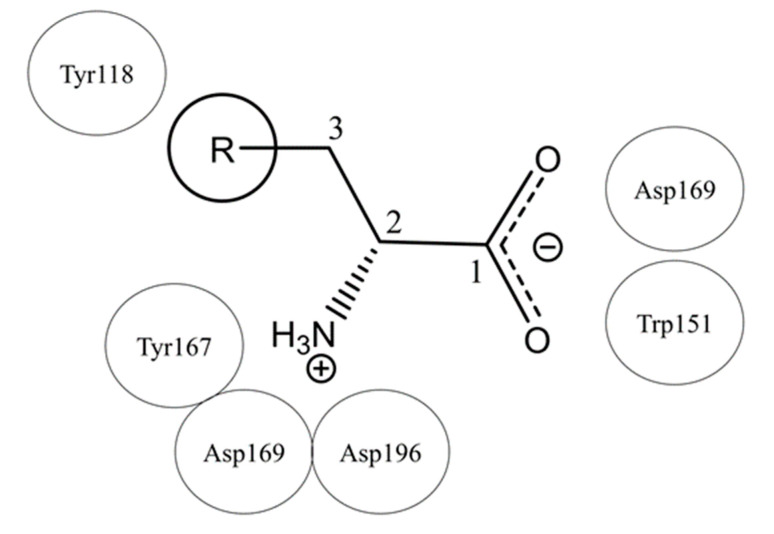
General structure of all *C. jejuni* Tlp3 ligands discovered in this study and in previous work [29,32]. R represents a hydrogen atom or a hydrophobic group. The protein residues that are indispensable for ligand binding are shown schematically next to the ligand moiety they interact with.

A previous site-directed mutagenesis study of Tlp3 LBD showed that the ammonium (-NH_3_^+^) and carboxylate (-CO_2_^−^) groups of the amino acid ligand form critical hydrogen bond interactions with the residues in the binding pocket of the membrane-distal subdomain of Tlp3 LBD [32]. In addition, the results of that study revealed that van der Waals interactions between the ligand side chain and the side chain of Tyr118 are also important, as the Tyr118Ala substitution resulted in at least a 35-fold reduction in the binding affinity of L-isoleucine [32]. The general structure of the new ligands discovered in this study and the abovementioned key interactions between Tlp3 and its ligands are presented schematically in Figure 9.

**Figure 10:**
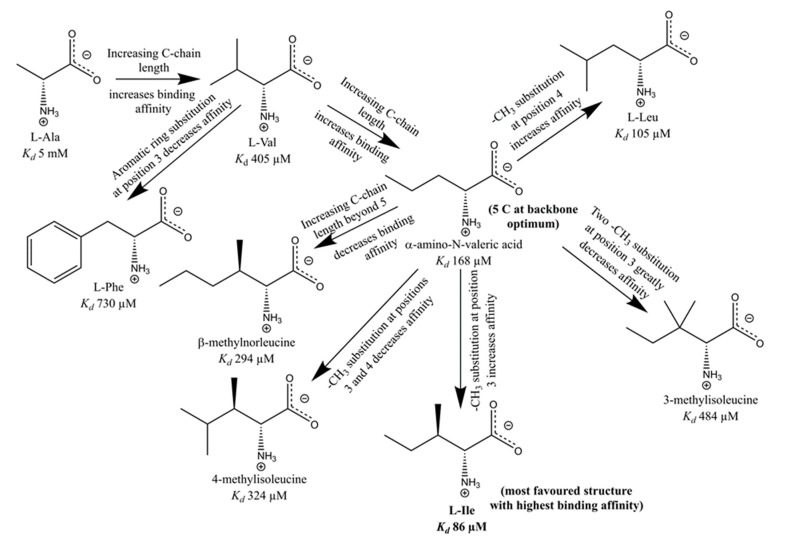
Structure–activity relationship of hydrophobic amino acids as chemoreceptor Tlp3 ligands.

The structure–activity relationship of hydrophobic amino acids as chemoreceptor Tlp3 ligands is depicted in Figure 10. L-alanine, the smallest known Tlp3 ligand, possesses the lowest binding affinity (*K*_d_ ≈ 5 mM). Increasing the length of the ligand’s linear carbon chain progressively increases the binding affinity (e.g., *K*_d_ (L-valine) = 405 µM), until an optimum length of five carbon atoms is achieved, as is the case with α-amino-N-valeric acid (*K*_d_ of 168 µM, Figure 10). Increasing the length of the carbon chain beyond five atoms decreases the binding affinity, as seen with β-methylnorleucine (*K*_d_ of 294 µM). Our crystallographic analysis provided a structural rationale for this phenomenon: the strongly hydrophobic side-chain-binding pocket can expand to accommodate molecules with the carbon backbone of up to five atoms in length, while longer linear side chains (such as that of β-methylnorleucine) are flipped to the outside of the pocket (Figure 7H), which reduces favourable van der Waals contacts with the protein and, hence, lowers the binding affinity. Methyl group (-CH_3_) substitutions along the carbon chain also greatly influence the binding affinity, and the effect depends on the position and the number of methyl groups (Figure 10). A single methyl group substitution at Position 3 increases the binding affinity more than the substitution at Position 4 (L-isoleucine (*K*_d_ = 86 µM) binds tighter than L-leucine (*K*_d_ = 105 µM)). However, substitution with two methyl groups at Position 3 results in a steric clash with the protein residues, reducing the binding affinity by at least 5-fold, as is the case with 3-methylisoleucine (*K*_d_ = 484 µM). Furthermore, simultaneous single methyl group substitution at Positions 3 and 4 has a deleterious effect on the binding affinity, as compared to single methyl substitutions at positions 3 or 4: 4-methylisoleucine displays a lower binding affinity (*K*_d_ = 324 µM) than L-isoleucine or L-leucine. Finally, aromatic ring substitution at Position 3 greatly reduces the binding affinity by introducing steric clashes with the protein atoms, as seen with L-phenylalanine (*K*_d_ = 730 µM). In summary, this analysis points to an amino acid with a five-carbon backbone and a methyl substitution at Position 3 (i.e., L-isoleucine) as the ligand with the optimal structure for a favourable interaction with Tlp3.

## 4. Discussion

Infection with *C. jejuni* is the most common bacterial cause of gastroenteritis in humans. Adding to the medical and economic costs are chronic post-infectious complications that include reactive arthritis and neuromuscular paralysis. With the rise of antibiotic resistance, the World Health Organisation flagged the urgent need for new anti-*C. jejuni* therapies in 2017 by listing fluoroquinolone-resistant *C. jejuni* as a high priority pathogen for antimicrobial research development. Chemotaxis, mediated by *C. jejuni* chemoreceptors, plays an important role in intestinal colonisation. Inactivation of the chemoreceptor Tlp3 reduces the ability of *C. jejuni* to invade human and chicken cells [27,29] and to colonise the jejunal mucosa of mice [30]. Chemical agents (e.g., inhibitors, antagonists) that target Tlp3 may, therefore, be useful for controlling *C. jejuni* infection. Knowledge of the structure of the ligand-binding domain of Tlp3 in complex with the signal molecules it senses is essential for the development of such therapeutics.

Tlp3 is an unusual chemoreceptor, in that it reportedly mediates chemotactic response to such chemically diverse molecules as isoleucine, purine, aspartic acid, malic acid, fumaric acid, sodium deoxycholate, lysine, glucosamine, succinic acid, arginine, and thiamine [29,30]. Controversially, signalling via direct binding to Tlp3 has been suggested for all these molecules, but lack of consistency across the results of different binding assays [29] has prompted us to put this hypothesis under scrutiny. By performing in vitro and in silico high-throughput screening for small molecules that bind to Tlp3 LBD, in conjunction with quantitative biophysical binding assays and X-ray crystallographic analysis, we firstly demonstrated that among all molecules listed above, only isoleucine signals through Tlp3 by binding directly to its ligand-binding domain (LBD). Our results imply that sensing of the remaining molecules is likely to be indirect, e.g., via periplasmic substrate-binding proteins.

We also discovered eight new small-molecule ligands that, in addition to isoleucine, signal through Tlp3 as attractants by binding directly to its LBD, and determined the crystal structures of the respective complexes. All new ligands (leucine, valine, α-amino-N-valeric acid, 4-methylisoleucine, β-methylnorleucine, 3-methylisoleucine, alanine, and phenylalanine) are nonpolar amino acids that are chemically and structurally similar to isoleucine. Detailed analysis of the structure–activity relationship in this series allowed us to identify molecular features recognised by this chemoreceptor. The hydrophobic side-chain binding pocket and the conserved protein residues that interact with the ammonium and carboxylate groups of the ligands determine the specificity of this chemoreceptor, which appears to have evolved as a receptor for natural non-polar amino acids. The binding pocket can contract and expand to allow binding of hydrophobic amino acids as small as L-alanine and as large as L-phenylalanine. Among the natural amino acids, the highest binding affinity was observed for isoleucine, followed by leucine, valine, phenylalanine, and alanine.

What is the physiological role of Tlp3-mediated taxis towards hydrophobic amino acids in *C. jejuni*? This pathogen has a dedicated import system for leucine, isoleucine, and valine, known as the LIV-I/LS (Leucine, Isoleucine, and Valine-I/Leucine specific) branched-chain amino acid ABC transporter system [76]. The LIV-I/LS system, comprising two periplasmic binding proteins LivJ and LivK, permeases LivH and LivM, and cytoplasmic ATPases LivG and LivF [76], also transports alanine [76,77] and phenylalanine [78], but to a lesser extent. Acquisition of these amino acids from the host may be necessary for bacterial protein synthesis. In addition, similar to many other Gram-negative bacteria, *C. jejuni* may use branched amino acids as precursors in the biosynthesis of diffusible signal factors – fatty acid derivatives that serve as quorum-sensing signal molecules [79]. *liv* mutant strains displayed a severe colonisation defect in mouse and chicken models [16,76]. The uptake of hydrophobic amino acids via this system, therefore, plays an important role in intestinal colonisation by *C. jejuni*, and our study suggests that *C. jejuni* seeks out hydrophobic amino acids using chemotaxis.

## Figures and Tables

**Table 1:** The top three L-isoleucine analogues identified as potential Tlp3 ligand-binding domain (LBD) ligands using an in silico screening.

ZINC ID	Chemical Name	Predicted Free Energy of Binding (kcal mol^−1^)	Aqueous Solubility at pH 7.4 (mM)
ZINC000001707292	4-methyl-L-isoleucine	−7.1	2030
ZINC000005841418	β-methyl-L-norleucine	−6.7	1743
ZINC000014967114	3-methyl-L-isoleucine	−7.2	61

**Table 2:** X-ray data processing statistics. Values in parentheses are for the highest resolution shell.

Dataset	Tlp3 LBD/ L-Leucine	Tlp3 LBD/ L-Valine	Tlp3 LBD/α-Amino-N-Valeric Acid	Tlp3 LBD/4-Methyl-L-Isoleucine	Tlp3 LBD/β-Methyl-L-Norleucine	Tlp3 LBD/3-Methyl-L-Isoleucine	Tlp3 LBD/L-Alanine	Tlp3 LBD/L-Phenylalanine
Space group	*P2*_1_	*P2*_1_	*P2*_1_	*P2*_1_	*P2*_1_	*P2*_1_	*P2*_1_	*P2*_1_
*a*, *b*, *c* (Å)	42.6, 137.5, 48.9	42.6, 137.5, 49.1	41.9, 137.8, 49.2	42.4, 137.7, 48.8	42.1, 138.1, 48.8	42.3, 138.1, 48.8	42.6, 137.6, 49.1	42.4, 137.4, 48.8
β (°)	94.3	94.5	93.9	94.0	94.7	93.8	94.7	94.0
Resolution range (Å)	28.21–1.40 (1.42–1.40)	28.18–1.40 (1.42–1.40)	46.23–2.10 (2.16–2.10)	40.45–1.42 (1.50–1.42)	35.86–1.38 (1.46–1.38)	35.99–1.38 (1.46–1.38)	46.12–1.32 (1.34–1.32)	45.85–1.38 (1.40–1.38)
R_merge_	0.048 (0.399)	0.047 (0.308)	0.077 (0.313)	0.042 (0.147)	0.056 (0.333)	0.068 (0.342)	0.026 (0.152)	0.032 (0.292)
Average *I*/σ(*I*)	10.7 (2.6)	11.4 (3.1)	10.6 (3.3)	17.1 (6.4)	10.1 (2.8)	9.1 (2.6)	15.9 (4.1)	12.4 (2.6)
Completeness (%)	99.2 (99.4)	94.3 (88.0)	97.6 (96.6)	98.5 (98.4)	89.1 (87.6)	81.1 (74.8)	97.5 (89.5)	98.7 (99.8)
Redundancy	3.7	3.2	4.3	3.6	3.1	3.5	3.5	3.2
Observed reflections	403, 905	328, 682	134, 763	375, 443	311, 496	320, 095	445, 181	354, 924
Unique reflections	109, 203	103, 884	31, 594	102, 980	100, 314	91, 649	128, 662	964, 43

**Table 3:** Refinement statistics.

Dataset	Tlp3 LBD/ L-Leucine	Tlp3 LBD/ L-Valine	Tlp3 LBD/α-Amino-N-Valeric Acid	Tlp3 LBD/4-Methyl-L-Isoleucine	Tlp3 LBD/β-Methyl-L-Norleucine	Tlp3 LBD/3-Methyl-L-Isoleucine	Tlp3 LBD/L-Alanine	Tlp3 LBD/L-Phenylalanine
R_work_/R_free_^a^	0.168/0.189	0.169/0.191	0.168/0.214	0.115/0.162	0.127/0.180	0.121/0.180	0.124/0.156	0.132/0.174
No. protein atoms	4465	4489	4221	4146	4186	4102	4207	4102
No. water molecules	820	944	241	936	845	833	799	758
Average *B* (protein atoms, Å^2^)	15	14	26	19	21	24	19	25
Average *B* (water molecules, Å^2^)	29	29	30	40	41	43	38	43
Average *B* (ligand, Å^2^)	10	10	15	17	20	18	14	25
r.m.s. deviations from ideality								
Bond lengths (Å)	0.005	0.005	0.006	0.012	0.012	0.012	0.012	0.012
Bond angles (°)	0.8	1.8	0.8	1.5	1.5	1.5	1.5	1.5
Ramachandran plot (%) Favoured	99	99	100	100	99	100	99	99
Allowed	1	1	0	0	1	0	1	1
Outliers	0	0	0	0	0	0	0	0

^a^ The R_free_ was calculated on 5% of the data omitted at random.

**Table 4:** Thermal shift assay data and thermodynamic parameters of ligand binding derived from isothermal titration calorimetry (ITC) measurements.

Compound	Thermal Shift	Thermodynamic Parameters		
	∆T_m_ (°C)	*K*_d_ (µM)	∆*H* (kcal mol^−1^)	∆*S* (cal mol^−1^ deg^−1^)
L-isoleucine ^a^	2.1 ± 0.8	86 ± 10	−4.4 ± 0.2	3.6
L-leucine	2.4 ± 0.2	105 ± 6	−4.6 ± 0.1	2.9
L-valine	1.3 ± 0.1	405 ± 27	−4.4 ± 0.2	0.8
α-amino-N-valeric acid	1.4 ± 0.3	168 ± 9	−6.5 ± 0.2	−4.5
Ala-Thr	1.1 ± 0.3	NB ^b^		
2-deoxycytidine	1.4 ± 0.8	NB		
methyl pyruvate	−1.8 ± 0.0	NB		
L-lysine	−0.5 ± 0.1	NB		
L-arginine	0.1 ± 0.2	NB		
L-aspartic acid	−0.2 ± 0.7	NB		
succinic acid	−0.1 ± 0.6	NP		
malic acid	−0.8 ± 0.4	NP		
thiamine	−0.1 ± 0.4	NP		
purine	NP ^c^	NB		
4-methyl-L-isoleucine	2.8 ± 0.3	324 ± 20	−5.1 ± 0.2	−1.01
β-methyl-L-norleucine	3.7 ± 0.2	294 ± 14	−6.2 ± 0.2	−4.77
3-methyl-L-isoleucine	2.0 ± 0.1	484 ± 30	−5.7 ± 0.2	−4.03
L-alanine	−0.6 ± 0.7	4980 ± 917	−150.2 ± 54.5	−495.50
L-phenylalanine	−0.3 ± 0.4	730 ± 55	−5.9 ± 0.3	−5.44

^a^*K*_d_ for L-isoleucine was determined in our previous study [32]; ^b^ NB: No binding detected by ITC measurements; ^c^ NP: Not performed.

## Supplementary Materials

The following are available online at https://www.mdpi.com/2218-273X/10/5/744/s1, Figure S1: Generation of isogenic *tlp3* mutant in *C. jejuni* NCTC 11168, Table S1: Tlp3 LBD thermal shift assay results with the compounds from the Biolog PM1, PM3B and PM5.
